# Prospective studies of infectious mononucleosis in university students

**DOI:** 10.1038/cti.2016.48

**Published:** 2016-08-12

**Authors:** Jennifer M Grimm, David O Schmeling, Samantha K Dunmire, Jennifer A Knight, Beth D Mullan, Julie A Ed, Richard C Brundage, Kristin A Hogquist, Henry H Balfour

**Affiliations:** 1 Department of Laboratory Medicine and Pathology, University of Minnesota Medical School, Minneapolis, MN, USA; 2 Department of Experimental and Clinical Pharmacology, College of Pharmacy, University of Minnesota, Minneapolis, MN, USA; 3 Department of Pediatrics, University of Minnesota Medical School, Minneapolis, MN, USA

## Abstract

We performed an intensive prospective study designed to obtain as much data as possible on the incubation and early illness periods of primary Epstein–Barr virus (EBV) infection. Undergraduate students who lacked EBV antibody and oral EBV DNA (EBV-naive) were seen every 2 weeks during their freshman year. Clinical and behavioral data, oral washes and venous blood were obtained. EBV antibodies were quantified by enzyme immunoassay and viral loads by PCR. During a median 8 months of observation, 14/85 subjects experienced primary EBV infections (24 cases/100 person-years). The only significant risk factor for acquisition of EBV infection was deep kissing (*P*=0.02). Eleven subjects had infectious mononucleosis with a median duration of 21 days. Two subjects were hospitalized. Infections were initially identified in 12 subjects by finding EBV DNA in oral cells before onset of symptoms and in 2 subjects by symptom reporting. EBV DNA and viral capsid antigen (VCA) IgM and gp350 IgG antibodies were present in the blood before onset of illness. To provide a more robust evaluation of primary EBV infection in undergraduate university students, we combined data on risk factors and antibody responses from this and an earlier study that used the exact same clinical and laboratory methods. The observation that the only significant risk factor for acquisition of EBV infection was deep kissing was confirmed. Most importantly, higher amounts of gp350 antibody correlated significantly with a lower severity of infectious mononucleosis (*P*<0.0001), which strengthens the rationale for a gp350-based prophylactic EBV vaccine.

Epstein–Barr virus (EBV) was discovered in 1964 by Epstein *et al.*^1^ using electron microscopy to detect the virus in cultured African Burkitt lymphoma cells. EBV has been documented to be one of the most common human viruses, infecting at least 90% of the worldwide population.^2^ Primary EBV infection can be completely asymptomatic or present as infectious mononucleosis.^3^ Risk factors for acquisition of primary EBV infection, the proportion that result in infectious mononucleosis, and the distribution of their severity can only be determined by prospective studies. These investigations are important for defining the burden of primary EBV infection, which will inform decisions on the appropriate use of a prophylactic vaccine, such as the gp350-based EBV vaccine reported to prevent infectious mononucleosis in a phase 2 clinical trial.^4^

Crawford *et al.*^5^ performed a prospective study on 241 EBV-naive students who enrolled in Edinburgh University in 1999 and 2000, and made follow-up visits ~3 years later. The incidence of primary EBV infection was 46% (110/241) during the 3-year observation period; 24.5% of infections (27/110) resulted in infectious mononucleosis. Significant risk factors for acquisition of EBV infection were genital contact with or without penetrative sexual intercourse. We conducted a prospective study of 143 EBV-naive students from the University of Minnesota Classes of 2010 and 2011 who enrolled as freshmen in 2006 and 2007.^6^ Their incidence of primary EBV infection during the 4-year observation period was 46% (66/143); 77% (51/66) had infectious mononucleosis. The only significant risk factor for acquisition of EBV infection was deep kissing with or without coitus.

Here, we report a new study of University of Minnesota undergraduate students from the class of 2016 who were seen every 2 weeks throughout their freshman year as compared with every 4–8 weeks in our previous investigation. The frequency of clinic visits enabled us to obtain more information on the incubation period and early acute illness than was available from our first study. The clinical and laboratory methods employed in our two studies were identical. Therefore, we believe that it is reasonable to present combined data on risk factors, viral loads and antibody responses to provide a more robust evaluation of primary EBV infection in undergraduate university students.

## Results

### Class of 2016 study

#### Prevalence and relative quantity of EBV antibody at screening

Of the 279 freshmen from the class of 2016 who were screened by enzyme immunoassay (EIA) for EBV VCA IgG antibodies, 144 (52%) were positive and 135 (48%) were negative. There were no equivocal antibody results. The median age of the subjects was 18.6 years (mean: 18.6; range: 18.0–21.1). The median EIA VCA IgG index for the antibody-positive subjects was 4.16 (mean: 3.95; range: 1.31–6.43).

#### Demographic factors associated with EBV antibody prevalence at screening

Antibody prevalence was higher among women (66 /112; 59%) than men (78/167; 47%) but the difference was not statistically significant. Students born outside the US had an antibody prevalence of 83% (10/12) as compared with 50% among US born students (134/267). Although this was statistically significant (*P*=0.04, Fisher's exact test 2-sided), the number of foreign-born subjects was small. There were differences in antibody prevalence by state of birth in the United States but these were not statistically significant (data not shown).

#### Prevalence of EBV DNA in oral washes at screening

EBV DNA was present in 29 (20%) of 142 oral washes from antibody-positive subjects at their screening visit. The median quantity among the positive subjects was 3900 copies per ml (mean: 15 400; range: 200–280 600). Of interest was that 3 (2%) of 135 VCA IgG antibody-negative students had EBV DNA in their oral wash (700, 9700 and 83 300 copies per ml, respectively). We performed additional testing on their screening samples and found that one of the three subjects had VCA IgM antibodies but none were heterophile-positive. These three subjects were not included in the surveillance phase because they had evidence of EBV infection before screening. Two of them subsequently developed infectious mononucleosis, whereas the third was lost to follow-up.

#### Surveillance subjects: demographics, clinic visits and samples tested

Of the 135 antibody-negative students, 89 (66%) enrolled in the surveillance phase and 85 (95.5%) of them completed it. These 85 included 49 men and 36 women who were similar to the group screened and the eligible pool of EBV-naive subjects in terms of age, sex, race/ethnicity and birthplace (data not shown). The surveillance cohort was 96.5% non-Hispanic white (82 subjects), 2.4% non-Hispanic black (2 subjects) and 1.2% Hispanic (1 subject). These 85 freshmen made 1307 clinic visits (median: 15 visits per subject; mean: 15.4; range: 11–22) at which interim medical and social data were collected. Overall, 1225 oral washes and 155 whole blood samples were tested for EBV DNA and 1086 EBV-specific antibody assays were performed, as well as 84 heterophile tests. The median duration of surveillance was 246 days (mean: 249; range: 182–296).

#### Incidence of primary EBV infection and how it was detected

Fourteen subjects (8 men and 6 women, 18–19 years old) experienced primary EBV infections during 58 person-years of observation. The incidence of primary EBV infection was 24 cases per 100 person-years at risk. The incidence was exactly the same in men and women.

Twelve of the 14 subjects who contracted primary EBV infections were called in for a ‘sick visit' because EBV DNA was detected in their oral cells for the first time. All of them had laboratory-confirmed primary EBV infection. At the time of the initial ‘sick visit,' 10 of the 14 also had EBV DNA in their oral supernatants and 6 had EBV DNAemia. Eleven of the 14 subjects developed infectious mononucleosis. The median time from detection of EBV DNA in the oral cell pellet until onset of symptoms was 6 days (mean: 5.8 days). Three of the 14 remained entirely asymptomatic.

Fifteen subjects were called into clinic because they reported symptoms consistent with infectious mononucleosis, but only two (13%) subsequently had laboratory-confirmed primary EBV infection. At the initial ‘sick visit,' both of these subjects had EBV DNA in their oral cell pellet and whole blood. Their clinical course was typical infectious mononucleosis.

#### Risk factors for acquisition of EBV infection

Alcohol consumption, energy level and stress were not risk factors but sexual behavior was. Subjects reporting deep kissing with or without coitus had very similar distributions of time to infection, and both groups were significantly different than the group of subjects who reported no kissing and no coitus (*P*=0.02).

#### Clinical findings

**Figure 1:**
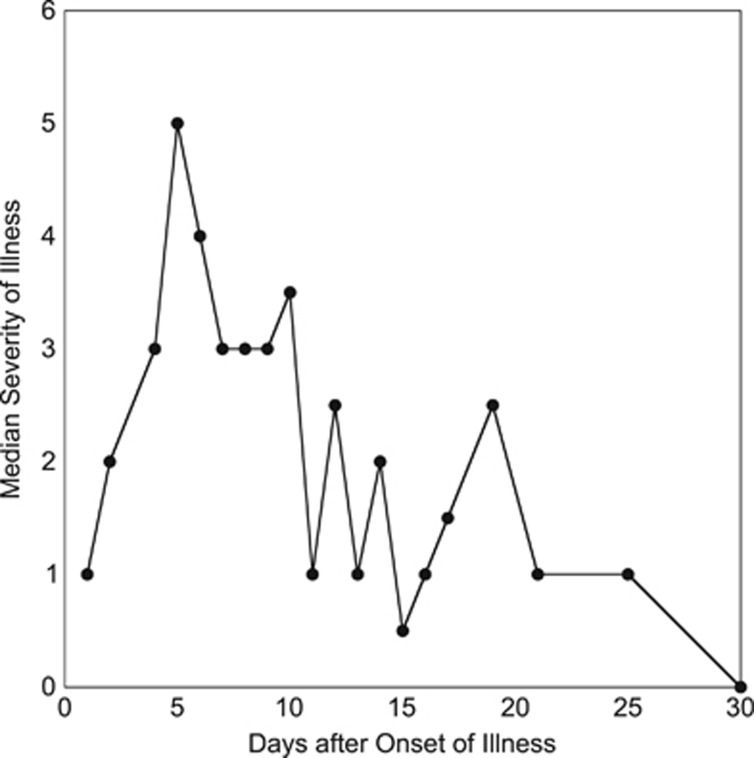
Median severity of illness scores during the month after onset of infectious mononucleosis among 11 subjects followed prospectively.

The clinical classification of primary EBV infection and the clinical findings are shown in Table 1. The individual maximum severity of illness (SOI) scores were: 0, 3 subjects; 3, 3 subjects; 4, 4 subjects, 5, 1 subject; 6, 3 subjects. The median maximum SOI score was 4 (mean: 4.4). The median SOI scores peaked 5 days after the onset of illness and then fluctuated downward to 0 after 30 days (Figure 1). The median duration of illness was 21 days (mean: 22.5; range: 10–43 days). Subjects were followed for a median of 196.5 days after acquisition of primary EBV infection (mean: 167; range: 57–281 days).

#### Viral loads and antibody responses during the incubation period of infectious mononucleosis

The incubation period was considered to be 42 days before the onset of symptoms of laboratory-documented infectious mononucleosis due to EBV based on previous reports^7, 8^ and our own research data.^9^ A total of 117 samples were collected from the 11 subjects with infectious mononucleosis during that time (Table 2). Ten of the 11 subjects (91%) had EBV DNA during the incubation period. All 10 had EBV in their oral cells. Virus was detected in the oral cells a median of 4 days before onset of illness. Seven of the 10 subjects with EBV DNA in their oral cells also had EBV in the oral supernatant and 4 had EBV DNA in whole blood. IgM antibodies against VCA and IgG antibodies against gp350 developed before the onset of illness in 4/9 and 5/9 subjects, respectively.

#### Viral loads, antibody responses and alanine aminotransferase (ALT) levels during primary EBV infection

All 14 infected subjects had EBV DNA in their oral cells, oral supernatant fluid and whole blood after the onset of primary EBV infection. The median duration of EBV DNA in the oral compartment could not be accurately calculated because 10/14 subjects were still positive at the final study visit, which was a median of 75 days after the onset of infection. The shortest documented duration of viral shedding was 28 days and the longest was 252 days. The median duration of EBV DNA in blood was 17 days and 1 subject had DNAemia for 164 days after the onset of infectious mononucleosis.

An EBV VCA IgM antibody response was documented in all 14 subjects, and these antibodies were present at the final visit (median, 91 days after onset of infection) in 7 subjects. Thirteen subjects developed EBV VCA IgG antibody. In symptomatic subjects, it was detected as early as 1 day before onset of symptoms and as late as 118 days after onset of symptoms. One subject was VCA IgG antibody-negative at the last study visit, but this was only 39 days after onset of symptoms. EBNA-1 antibody responses developed in 9 of the 14 subjects. All 3 asymptomatic subjects developed EBNA as compared with 6 of 11 subjects with infectious mononucleosis.

Heterophile antibodies were documented in 13 (93%) of the 14 subjects with primary EBV infection. They were first detected a median of 2 days after onset of illness and remained positive in 7 subjects at the last study visit (median, 143 days after onset of infection).

A total of 63 ALT levels were performed on the 14 subjects during their primary EBV infection. ALT levels were elevated in all 11 subjects with infectious mononucleosis and in 1 of 3 asymptomatic subjects. Elevated levels were first detected a median of 6 days after onset of illness and remained elevated for a median of 14 days. The median peak level was 153 U l^−1^ (range: 96–492).

### Combined data from the class of 2016 and classes of 2010–11 studies

**Figure 2:**
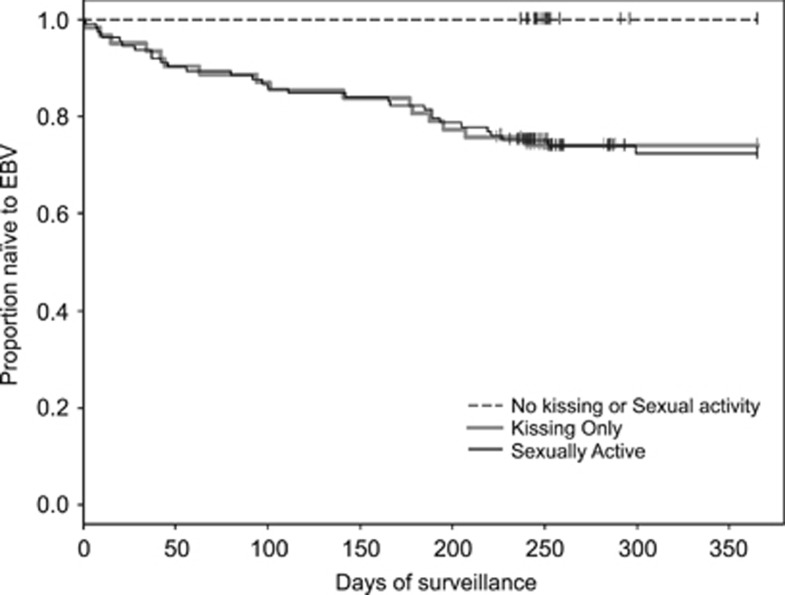
Social history data and acquisition of primary EBV infection during freshman year for the classes of 2010, 2011 and 2016. Information was provided by 203 (89%) of 228 subjects. The difference in the rate of acquisition of primary EBV infection between the ‘No Kissing or Sexual Activity' group (*N*=30) was significantly different than the ‘Kissing Only' (*N*=60) or ‘Sexually Active' (*N*=113) groups. *P*=0.0003, log-rank test.

The clinical and laboratory methods employed in the two studies were identical, and thus we considered it reasonable to compare and combine data on risk factors, viral loads and antibody responses to provide a more robust evaluation of primary EBV infection. The only significant risk factor for acquisition of EBV infection in both studies was deep kissing, which became more robust when the studies were combined (Figure 2).

**Figure 3:**
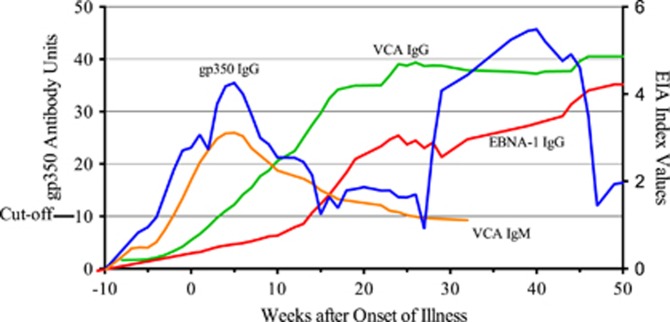
EBV-specific antibody responses among 80 subjects with primary EBV infections followed prospectively. Values above the cutoff are considered positive. Curve smoothing was performed using Prism software, averaging the closest four neighbors of a single point. IgG antibody against gp350 and IgM antibody against VCA were first to appear and then waned. The gp350 response was biphasic with the highest peak occurring 40 weeks after onset of illness. IgG antibody against EBNA-1 was the slowest to appear but remained elevated as did IgG antibody against VCA.

**Figure 4:**
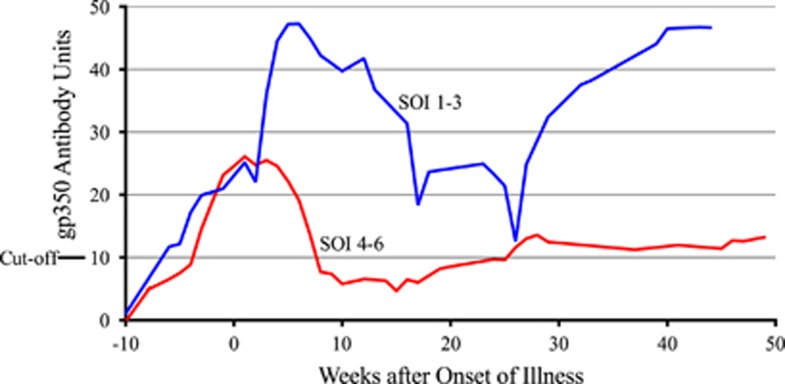
gp350 IgG antibody levels in 27 subjects who had infectious mononucleosis with at least four samples collected during the first year after onset of illness. Values above the cutoff are considered positive. The area under the AUC was significantly greater among 12 subjects whose maximum SOI was milder (maximum SOI score 1–3) as compared with 15 subjects whose illness was more severe (maximum SOI score 4–6). AUC, 1641 versus 661; *P*<0.0001, unpaired, two-tailed *t*-test.

Combined EBV-specific antibody responses are shown in Figure 3. IgM antibody against VCA appeared rapidly but then waned. IgG antibody against EBNA-1 was the slowest to appear, but remained elevated as did IgG antibody against VCA. The gp350 IgG antibody response was biphasic with the highest peak occurring 40 weeks after onset of illness. The amount of gp350 IgG antibody produced correlated with less severe infectious mononucleosis. The area under the antibody quantity–time curve (AUC) was significantly greater among 12 subjects whose maximum SOI was milder (maximum SOI score 1–3) as compared with 15 subjects whose illness was more severe (maximum SOI score 4–6). AUC, 1641 versus 661; *P*<0.0001, unpaired, two-tailed *t*-test (Figure 4). This was not the case for VCA IgM, VCA IgG and EBNA-1 antibodies (data not shown).

Considering viral loads, all 14 subjects in the class of 2016 study had EBV DNA in the blood as compared with 42 of 65 (65%) in the classes of 2010–11 study. Also, the duration of EBV DNAemia was longer in the class of 2016 study (median: 17 versus 1 day). All subjects in both studies had EBV DNA in the oral compartment. Levels peaked 2 months after onset of illness with a median duration of oral shedding of 5.2 months. The shortest documented period of oral shedding was 28 days and the longest was 3.1 years.

Overall, 79 of 80 subjects with primary infections had samples available for EBNA typing. EBV EBNA type 1 was present in 69 subjects (87%), EBNA type 2 in 8 subjects (10%) and mixtures of the 2 types in 2 subjects (2.5%). The distribution of EBNA types was similar in both studies: 93% were type 1 in the class of 2016 study and 90% were type 1 in the classes of 2010–11 study. There was no correlation between disease severity and EBNA type (data not shown).

## Discussion

An important finding was that the quantity of gp350 antibody produced over time inversely correlated with the severity of disease. In other words, subjects with greater amounts of antibody had milder cases of infectious mononucleosis. This implies that gp350 antibody could be protective and strengthens the rationale for a prophylactic gp350-based EBV vaccine.^4^ In contrast to our finding, Bu *et al.*^10^ did not observe a correlation between peak levels of gp350 antibody and clinical severity. However, their samples did not include subjects from our class of 2016 study, and they did not report an AUC analysis to quantitate the antibody response.

The present study confirms our previous observation that deep kissing with or without penetrative sexual intercourse is the major risk factor for acquisition of primary EBV infection. Combined sexual history data from 203 freshmen clearly establishes this point (Figure 2).

The class of 2016 subjects were sicker (median SOI score 4 versus 3) for a longer period of time (median duration of illness, 21 days versus 10 days) than the students in the classes of 2010–11. This could have happened by chance, but it also could reflect pathogenic differences among EBV strains circulating during a particular year. It was not due to a difference in EBNA types because both studies had similar frequencies of EBNA type 1 and type 2.

Our prospective studies have documented that the incidence of primary EBV infection during the four undergraduate years was 14.4 cases per 100 person-years. This rate is similar to the rate of 15.2 cases per 100 person-years reported by Crawford *et al.*^5^ among undergraduates at Edinburgh University, Scotland. However, our data differ from the Scottish study in that 77.5% (62/80) of our students developed infectious mononucleosis after primary EBV infection as compared with only 24.5% (27/110) among the Edinburgh University students. The reason for this difference is not clear. Our higher rate of infectious mononucleosis could be due to more frequent observations, differences in the genetic background and environment of the study populations or variations in the virulence of strains circulating during a particular year as mentioned above.

Subjects in the class of 2016 investigation were seen nine times during the 6 weeks after the diagnosis of primary EBV infection versus an average of two visits for the classes of 2010–11. Such closely-spaced clinic visits with blood sampling permitted us to document DNAemia in all 14 infected class of 2016 subjects as compared with 42 of 65 (65%) subjects in our earlier study. It also allowed us to better characterize the duration of viremia, which was a median of 17 days. The median duration of 1 day reported for the earlier study undoubtedly resulted from infrequent sampling during the acute illness.

EBV DNA can be found in the oral cavity and blood before the onset of symptoms of primary EBV infection. Indeed, 12 of the 14 primary EBV infections were first detected by obtaining a positive oral wash from subjects who were still asymptomatic. This observation was possible because oral washes were obtained every 14 days and tested no later than 4 days after collection. Subjects with primary EBV infection were continuously positive for oral EBV DNA for at least 4 weeks, all were viremic, and all developed EBV-specific antibodies.

Our group has described the viral and cellular immune events during the incubation period of primary EBV infection among 40 subjects from this study and our previous prospective clinical investigation.^11^ Using a qualitative nested PCR method, EBV DNA was found in the blood about 3 weeks before onset of illness, whereas EBV DNA was not detected in the oral compartment until a week before symptoms developed. These data differ from what is reported here, namely that EBV DNA was found in oral cells a median of 4 days before illness and in blood a median of 2.5 days before symptoms (Table 2). This difference is most likely due to the increased sensitivity of the qualitative PCR as compared with the quantitative PCR method employed in the class of 2016 study.

Liver involvement is very common in infectious mononucleosis but is usually documented by elevation of liver enzymes without signs or symptoms of hepatitis.^12^ By performing serial ALT measurements, we found that 12 (86%) of our 14 subjects with primary EBV infection had elevated ALT values. ALT levels were elevated in all 11 symptomatic subjects. None of our subjects was jaundiced or had hepatic tenderness but 4 of them reported abdominal pain, which could have been due to liver inflammation.

The prevalence of EBV antibody among entering freshmen students at the University of Minnesota declined between 2006 and 2012, which is consistent with data from the National Health and Nutrition Examination Surveys (NHANES). In NHANES, EBV antibody prevalence among US children 6–19 years old declined from an average of 72% during 2003–2004 to 65% during 2009–2010.^13^ EBV VCA IgG antibody prevalence for University of Minnesota students who entered college in 2006, 2007 and 2012 was 64, 62 and 52%, respectively. The decline in antibody prevalence averaged 2% annually. The explanation for this is not clear but it has also been observed in Japanese children.^14^ If this trend continues, the number of adolescents and young adults who are most susceptible to infectious mononucleosis will increase.

By performing oral washes on all subjects who were screened, we found that the prevalence of asymptomatic oral shedding among VCA IgG antibody-positive 18-year-old freshmen was 20% (29/142). This was actually lower than the oral shedding prevalence of 39% (34/87) that we reported for asymptomatic adults ages 20–64 (mean age: 34) who participated in a 6-month surveillance study.^15^ An unexpected finding was that 3 (2%) of 135 VCA IgG antibody-negative students had EBV DNA in their oral wash. This has caused us to redefine EBV-naive as individuals negative for both VCA IgG antibodies and oral EBV DNA.

Infectious mononucleosis is not a trivial illness especially for university students. Macsween *et al.*^16^ reported that the disease ‘resulted in marked reductions in student study time, physical exercise and non-exercise-related social activities' among undergraduates at Edinburgh University. Two of their 56 students with infectious mononucleosis dropped out of school completely, and three had to repeat a year of study. In the class of 2016 study, the median duration of illness was 21 days, and 2 of the 14 subjects with primary EBV infections were hospitalized. The Scottish and University of Minnesota studies underscore the potential benefit of a prophylactic EBV vaccine. Our finding that subjects who produced more gp350 antibody had less severe cases of infectious mononucleosis strengthens the rationale for a gp350-based EBV vaccine.

## Methods

### Clinical study design

Both the class of 2016 study and the classes of 2010–11 study had a screening and a surveillance phase.

### Screening phase

University of Minnesota freshmen who gave informed consent completed a demographic/history form and donated 10 ml of venous blood and an oral wash. Information collected included birthdate, birthplace and race/ethnicity. Plasma samples and oral washes were tested as described below. Results were discussed with the students and those who were EBV-naive, defined as lacking EBV VCA IgG antibodies by EIA, were invited to participate in the surveillance phase.

### Surveillance phase

Subjects in the surveillance phase made visits to the research clinic every 2 weeks for the class of 2016 study and every 4–8 weeks for the classes of 2010–11 study while school was in session. Subjects in the 2016 study were followed only during their freshman year, whereas those in the class of 2010–11 study were followed for all four undergraduate years. At each visit a medical history, oral wash and health questionnaire were obtained. Questions included information on sexual behavior, alcohol consumption, energy level and stress. At every other visit, 40 ml of venous blood was drawn. Surveillance samples were tested for EBV antibodies and viral DNA as soon as possible but no longer than 4 days after collection.

### Ethics statement

Both studies were approved by the Research Subjects Protection Program of the University of Minnesota, and subjects gave written informed consent before participation.

### Identification of primary EBV infection

The methods for collection of clinical data and identification of primary EBV infection were exactly the same for the class of 2016 study and the classes of 2010–11 study. Subjects were instructed to report signs and symptoms suggestive of an acute EBV infection by e-mail or by phone. They were seen in the research clinic as soon as possible after that for a ‘sick visit,' which consisted of a physical examination, oral wash and a blood draw. Samples were tested immediately for EBV antibody and EBV DNA as described below. Subjects who did not report symptoms but whose surveillance samples became positive for EBV DNA and/or EBV antibodies were seen as soon as possible for a physical examination, oral wash and a blood draw.

Primary EBV infection was initially identified as the presence of EBV DNA in the oral and/or blood compartment of a subject who was previously negative for both EBV antibodies and EBV DNA. It was confirmed by documenting continuous oral shedding and the development of EBV-specific antibodies. Primary EBV infection was classified clinically as infectious mononucleosis (at least two of the following: sore throat, cervical lymphadenopathy, fever, fatigue); symptomatic (but not fulfilling the definition of infectious mononucleosis) or asymptomatic. The SOI was evaluated using a categorical scale from 0 (asymptomatic) to 6 (essentially bedridden) as previously published.^6, 17^ In the class of 2016 study, subjects who met the criteria for primary EBV infection were seen twice a week for 3 weeks, and then once a week for the next 3 weeks for a total of 9 primary infection visits. They continued to be followed after that every 2 weeks until the end of their freshman year. In the classes of 2010–11 study, subjects who acquired a primary EBV infection were seen at least twice during the acute illness and then every 8 weeks thereafter.

EBV DNA was quantified in the oral and blood compartments, and EBV VCA IgM, VCA IgG and EBNA-1 IgG antibodies were performed at every visit postinfection. Subjects were also tested periodically until their final study visit for heterophile antibodies, gp350 IgG antibodies and alanine aminotransferase levels.

### Sample collection and laboratory assays

The oral wash procedure, detection and quantitation of EBV DNA, and EIA assays for EBV VCA IgM, VCA IgG, EBNA-1 IgG and heterophile antibodies were performed as previously described.^6, 17^ Antibodies against gp350 were measured using an EIA developed by Servat *et al.*^18^ with minor modifications. The linearity of all antibody assays for both the instrument and subject samples were evaluated for *R*^2^ values using a prepared dye solution as recommended in the Biotek Spectrophotometer Manual (Bio Tek Instruments Inc, Highland Park, VT, USA). Absorbance was tested using the prepared dye solution of known absorbance in 10-fold dilutions from 10^0^ to 10^−10^. Subject samples, run in triplicate to assess precision, were tested using twofold dilutions from undiluted to 1:128. Graph plots of the data showed good fit and *R*^2^ values for all tests were between 0.992 and 0.998.

EBNA typing was performed with EBNA-2 primers that were described by Higa *et al.*^19^ with minor modifications.

ALT levels were determined by a bi-chromatic rate measurement assay using a Siemens Dimension Xpand Plus Analyzer (Siemens Healthcare Diagnostics, Brookfield, CT, USA). The normal values are 0–50 for persons <20 years old.

### Statistics

Statistical analysis was performed using Prism software (GraphPad Software, Inc, La Jolla, CA, USA), SAS University Edition (SAS Institute, Cary, NC, USA) or R (R Foundation for Statistical Computing, Vienna, Austria). Comparisons between groups were performed with two sample two-tailed *t*-tests for continuous outcomes and with χ^2^ for categorical outcomes. Spearman-rank correlation coefficient was calculated to assess associations. Baseline characteristics were assessed as risk factors by a χ^2^ test and by comparing Kaplan–Meier estimates of time to EBV infection with a log-rank test. VCA IgM, VCA IgG, EBNA-1 IgG and gp350 IgG antibodies at weeks post onset of illness of subjects with a maximum SOI score between 1 and 3 were compared with those of subjects having a maximum score between 4 and 6. Curve smoothing was completed by taking the closest four neighbors to a single point and averaging them. A column analysis unpaired *t*-test was performed using the normalized values from this smoothing to compare the mean gp350 antibody units of these two groups. An *f*-test for variance was also considered when analyzing these data. The area under the curve was calculated by setting a baseline at *y*=0.

## Figures and Tables

**Table 1:** Clinical classification and clinical findings in primary EBV infection

*Characteristic*	*Proportion positive (percent)*
*Clinical classification*^a^	
Infectious mononucleosis	11/14 (79%)
Symptomatic, not mono	0/14 (0%)
Asymptomatic	3/14 (21%)
	
*Clinical findings*	
Sore throat	11/11 (100%)
Cervical lymphadenopathy	11/11 (100%)
Fatigue	11/11 (100%)
Decreased appetite	11/11 (100%)
Headache	9/11 (82%)
Fever	8/11 (73%)
Upper respiratory symptoms	8/11 (73%)
Body aches	8/11 (73%)
Abdominal pain	4/11 (36%)

a Infectious mononucleosis (at least two of the following: sore throat, cervical lymphadenopathy, fever, fatigue); symptomatic (but not fulfilling the definition of infectious mononucleosis) or asymptomatic.

**Table 2:** EBV DNA and EIA antibody responses during the incubation period of infectious mononucleosis

*EBV assay*	*Proportion positive (%)*		*Median day positive before onset of symptoms (range)*	*Median log10* *copies per ml* *or relative antibody units (range)*^a^
	*Subjects*	*Samples*		
*Quantitative DNA*				
Oral cell pellet	10/11 (91)	13/31 (42)	4.0 (1–8)	4.19 (3.18–6.00)
Oral supernatant	7/11 (64)	9/31 (29)	4.0 (2–8)	2.30 (2.30–5.15)
Whole blood	4/9 (44)	5/19 (26)	2.5 (1–8)	2.60 (2.48–4.48)
				
*EIA antibodies*				
VCA IgM	4/9 (44)	6/18 (33)	1.5 (1–36)	2.50 (1.64–5.25)
VCA IgG	0/9 (0)	0/18 (0)	—	—
gp350 IgG	5/9 (56)	7/13 (54)	22 (4–39)	38.9 (13.6–85.4)

Abbreviations: EIA, enzyme immunoassay; VCA, viral capsid antigen.

a Of positive samples.
